# Allergenic Lipid Transfer Proteins from Plant-Derived Foods Do Not Immunologically and Clinically Behave Homogeneously: The Kiwifruit LTP as a Model

**DOI:** 10.1371/journal.pone.0027856

**Published:** 2011-11-17

**Authors:** Maria Livia Bernardi, Ivana Giangrieco, Laura Camardella, Rosetta Ferrara, Paola Palazzo, Maria Rosaria Panico, Roberta Crescenzo, Vito Carratore, Danila Zennaro, Marina Liso, Mario Santoro, Sara Zuzzi, Maurizio Tamburrini, Maria Antonietta Ciardiello, Adriano Mari

**Affiliations:** 1 Center for Molecular Allergology, IDI-IRCCS, Rome, Italy; 2 Institute of Protein Biochemistry, CNR, Naples, Italy; Dana-Farber Cancer Institute, United States of America

## Abstract

**Background:**

Food allergy is increasingly common worldwide. Tools for allergy diagnosis measuring IgE improved much since allergenic molecules and microarrays started to be used. IgE response toward allergens belonging to the same group of molecules has not been comprehensively explored using such approach yet.

**Objective:**

Using the model of lipid transfer proteins (LTPs) from plants as allergens, including two new structures, we sought to define how heterogeneous is the behavior of homologous proteins.

**Methods:**

Two new allergenic LTPs, Act d 10 and Act c 10, have been identified in green (*Actinidia deliciosa*) and gold (*Actinidia chinensis*) kiwifruit (KF), respectively, using clinically characterized allergic patients, and their biochemical features comparatively evaluated by means of amino acid sequence alignments. Along with other five LTPs from peach, mulberry, hazelnut, peanut, mugwort, KF LTPs, preliminary tested positive for IgE, have been immobilized on a microarray, used for IgE testing 1,003 allergic subjects. Comparative analysis has been carried out.

**Results:**

Alignment of Act d 10 primary structure with the other allergenic LTPs shows amino acid identities to be in a narrow range between 40 and 55%, with a number of substitutions making the sequences quite different from each other. Although peach LTP dominates the IgE immune response in terms of prevalence, epitope recognition driven by sequence heterogeneity has been recorded to be distributed in a wide range of behaviors. KF LTPs IgE positive results were obtained in a patient subset IgE positive for the peach LTP. Anyhow, the negative results on homologous molecules allowed us to reintroduce KF in patients' diet.

**Conclusion:**

The biochemical nature of allergenic molecule belonging to a group of homologous ones should not be taken as proof of immunological recognition as well. The availability of panels of homologous molecules to be tested using microarrays is valuable to address the therapeutic intervention.

## Introduction

Allergic disease prevalence is on the increase worldwide, and recent reports show that food allergy is going to be a greater problem than before [1]. Thus there is the need for a robust understanding of all aspects characterizing IgE response to allergens, being the IgE production the first step for allergy-mediated food hypersensitivity [2]. A great help in the process of a better knowledge in the field is coming from the increasing number of allergenic molecules identified so far (http://www.allergome.org/script/statistic.php) and made available for studies in combination with micro-technology [3]. Such combination allows exploring in deep details relationships among structurally distant as well as closely related homologous molecules [4].

The grouping of allergens in families because of their biochemical structure is leading to assign a similar IgE immune-recognition to similar structures, furthermore supported by *in silico* studies [5]. Allergenic molecules are in fact currently considered to be a unique entity because of their biochemical definition [6], [7]. That is the case of many allergen families, which got the definition of panallergens because of their distribution in certain distant subsets of living organisms rather than for their real and obvious IgE pan-recognition of protein structures [8]. Recent reports based on broad IgE testing using the powerful combination mentioned above show how the IgE-mediated immune response toward homologous structure could be influenced by other factors not easily interpreted just using the molecule structures [4].

Nowadays there is a claim for a more personalized medicine. Such claim seems to fit perfectly in the allergy field where the clinical phenotype is tightly linked to the IgE immune recognition and each patient seems to display a different clinical picture when compared to another. Such personalized approach, formerly almost impossible because of the number of tests to be performed in each patient, is now becoming increasingly feasible because of the biotechnology/microtechnology combination supported by information technology tools recently made available for the routine work [3], [9].

Plant LTPs are widely distributed, structurally related, small proteins involved in defense mechanisms. Although their lipid-binding ability has been well reported, the biological function of LTPs is still largely unknown. The plant LTP family includes two subfamilies according to their molecular masses: the 9-kDa LTP1 and the 7-kDa LTP2. Although LTP1 and LTP2 share a common compact fold consisting of four α-helices stabilized by four disulfide bridges, the pairing partners of cysteines are not completely conserved between the two subfamilies, that also display a low overall sequence similarity (about 30% identity) [10].

Up to now 63 LTPs have been characterized as allergens, being 46 of them expressed in edible parts of plants, almost all of them belonging to the LTP1 protein subfamily, and just two, having very preliminary reported data, to the LTP2 subfamily (www.allergome.org, accessed September 12, 2011).

Several reviews on the topic of LTP as allergens reported preliminary evidence of a heterogeneous behavior of this group of molecules [11]–[13], but few of them suggested strategies how to overcome the peculiarity of such behavior within a routine workup [13]. Unless we performed a broad study on 23,077 subjects using the microarray approach, we could not have evidence of such heterogeneity as we had just the LTP from peach available on the microarray used at the time the study had been performed [4].

Taking advantage of the implementation of a full molecule-, microtechnology-, and information technology-based infrastructure at the Center in Rome, Italy, and of the expansion of the number of available LTPs to a panel of seven allergens, including two new proteins identified in the kiwifruits (KF), we sought to compare the LTP biochemical, immunochemical, and clinical features in order to define the extent of their heterogeneity. The study, leading first to the full characterization of KF LTPs, brought us to abandon the former interpretation of “one molecule fits all” and to search for as much differences as possible among allergenic proteins facing each allergic patient, in order to increase the quality of personal decision making he or she deserves.

## Materials and Methods

### Allergic subjects

The study received the approval of the Institutional ethical committee of the Istituto Dermopatico dell'Immacolata, Rome, Italy (66/CE/2006). Patients or caregivers signed an informed consent when undergoing tests not in the routine workup. Patients' demographical and clinical data, namely respiratory and ingestion-related symptoms on KF exposure, as well as all the in vivo and in vitro diagnostic data, were recorded for all patients by an allergy specialist, or transferred real time from the laboratory, into the InterAll software, a customized allergy electronic record for diagnostic and clinical data storing (version 3.0, Allergy Data Laboratories s.c., Latina, Italy). KF specific clinical information were collected using the standard questionnaire reported in a previous study [14]. Patients who underwent clinical tests, like the skin test (ST) or the double blind placebo controlled food challenge (DBPCFC), were selected for any given study procedure following criteria reported in the Result section.

A subset of 259 consecutive subjects, tested nPru p 3 positive on the Immunosolid phase Allergen Chip (ISAC) 103 (Phadia Multiplexing Diagnostics, PMD, Vienna, Austria), was tested on the ISAC Exp96 (PMD, see below for details). To verify the presence of LTP IgE positive subjects beside the nPru p 3 in the subset reported above, a control population of 744 consecutive allergic subjects tested positive to any allergen on ISAC 103 has been created. Overall 1,003 subjects underwent IgE testing with the ISAC Exp96 microarray.

### Clinical testing

ST were performed and recorded as wheal areas using a standard methodology as already reported [14]. The following preparations were used: a commercial KF extract (Stallergenes, Antony, France), fresh KFs tested by means of the prick-prick technique, the green KF pulp and seed preparations, natural (n) purified Act d 10, nAct c 10, and nPru p 3. Details on the last five preparations are given below.

KF and peach DBPCFC have been performed as reported in literature in enrolled subjects when they did not report a recent anaphylaxis episode [14], [15]. In case of patients reporting recent anaphylaxis after KF or peach ingestion, it was taken as a proof of clinical allergy in accordance with the most recent European guidelines for the diagnosis of clinically relevant food allergy [16].

### Laboratory testing

#### Singleplexing IgE detection

Total and specific IgE using a singleplex system were determined in patients' sera from venous blood samples obtained at the time of enrolment by the ImmunoCAP system (Phadia AB, Uppsala, Sweden). To verify the IgE binding capability of the new KF LTPs, biotinylated nAct d 10 and nAct c 10 (see below) have been coupled on a streptavidin-CAP following the standard methodology as suggested by the manufacturer (Phadia) and reported in literature [17].

#### Multiplexing IgE detection

Specific IgE for allergenic molecules were detected by ISAC 103 microarray test (PMD); ISAC tests were performed as previously reported [4]. A customized version of the ISAC microarray (ISAC Exp96) has been developed to carry out the characterization of the IgE reactivity toward the new KF LTPs and some others not available on the commercial ISAC 103. For the purpose of the present study, data on nPru p 3, the peach LTP, recombinant ® Cor a 8, the hazelnut LTP, and nArt v 3, the mugwort pollen LTP, were obtained using the commercial ISAC 103 microarray. rAra h 9, the peanut LTP, provided by Phadia, and nAct d 10, nAct c 10, nMor n 3, the mulberry LTP, and the nPru p 3 in-house preparations were immobilized on ISAC Exp96 microarray, following the same methodology as for the routine ISAC 103 [18]. Details on each allergen biochemical, immunological, and clinical features are available via the Allergome web site (www.allergome.org) [9].

#### Single Point Highest Inhibition Achievable assay (SPHIAa)

IgE inhibition experiments have been performed applying the SPHIAa as previously reported [14]. Briefly, 20 µl of individual patients' sera have been incubated overnight with 20 µl of a solution containing the highly purified allergen preparation of nAct c 10, nAct d 10, nPru p 3, at 1 mg/ml concentration, or the simulated gastric fluid (SGF) or the trypsin digested preparations at the same concentrations. Whole KF, pulp and seed extract preparations (see below) were used at 1 mg/ml concentration considering their total protein content. All the above preparations acted as inhibitors of the IgE binding. After o.n. incubation, the IgE binding inhibition was evaluated by running the ISAC Exp96 microarray (PMD) where the IgE reactivity was evaluated on all immobilized nAct c 10, nAct d 10, rAra h 9, nArt v 3, rCor a 8, nMor n 3, and nPru p 3 at the same time, or on some of them depending on the experiments as described in details in the Results section. A control sample, where only the buffer solution was added, was used as reference value for no IgE inhibition. A trypsin solution has been added to controls when the SPHIAa using digested preparations was performed. To control the specificity of the IgE inhibition obtained on LTP molecules, several other allergens available on the two microarrays which were recognized by the IgE of the same patient or the pool were used. A no-inhibition value on those allergens was required to score the experiment as valuable. All IgE inhibition data were stored in the InterAll database, and percent inhibition values were calculated real time by a specific procedure developed within the InterAll software.

### Preparation of the protein extracts from whole kiwifruit

Gold KF (*Actinidia chinensis*) and green KF (*Actinidia deliciosa*) were purchased at a local market. KFs were peeled, homogenized in a household blender after addition of 1 M NaCl (1∶1 w/v) and stirred at 4°C for 2 h. After centrifugation at 12,500 x *g* for 60 min, the supernatant representing the total protein extract was collected, dialyzed against water, aliquoted and stored at −20°C until used.

### Preparation of protein extracts from pulp and from seeds

Seeds of gold and green KFs were manually separated from the pulp. Pulp samples were homogenized in a blender after addition of 1 M NaCl (1∶1 w/v) and stirred at 4°C for 2 h. Seeds were crushed by pestle in a mortar until a smooth powder was obtained. Seed proteins were extracted in 0.5 M NaCl, at 4°C for 2 h. After protein extraction, pulp and seed samples were centrifuged at 12500 x *g* for 60 min and the supernatants were collected.

### Determination of the protein concentration

The protein concentration in the extracts was estimated by the BIO-RAD Protein Assay (Bio-Rad, Milan, Italy), using calibration curves made with bovine serum albumin.

### Protein extracts fractionation by RP-HPLC

RP- HPLC of KF extracts was performed on a Vydac (Deerfield, IL, USA) C8 column (0.21×25 cm), using a Beckman System Gold apparatus (Fullerton, CA, USA). Elution was accomplished by a multistep linear gradient of eluant B (0.08% TFA in acetonitrile) in eluant A (0.1% TFA) at a flow rate of 1 ml/min. The eluate was monitored at 220 and 280 nm. The separated fractions were manually collected and analyzed.

### Purification of LTP from gold and green kiwifruits

KF LTPs, namely nAct c 10 and nAct d 10, were extracted from seeds and purified from the seed extracts using the procedure described by Ciardiello et al. [19]. nPru p 3 and nMor n 3 were purified from peach peel and from the whole black mulberry fruit, respectively, as previously reported [19]. Purity of the protein preparations was checked by SDS-PAGE, RP- HPLC and N-terminal amino acid sequencing.

### Molecular mass of purified LTPs

The molecular mass of purified proteins was estimated by MALDI-TOF mass spectrometry measurements carried out on a PerSeptive Biosystems (Framingham, MA, USA) Voyager-DE Biospectrometry Workstation. Analyses were performed on pre-mixed solutions prepared by diluting samples (final concentration 5 pmol/ml) in 4 volumes of matrix, namely 10 mg/ml α-ciano-4-hydroxycinnamic acid in 60% acetonitrile containing 0.3% TFA.

### Treatment of purified LTPs with SGF and trypsin

In vitro simulated gastric digestion of nAct d 10 and nPru p 3 was performed as described by Bublin et al. [20]. LTPs were subjected to pepsin digestion (Roche Diagnostics GmbH, Germany) using an enzyme/substrate ratio of 1: 20 w/w. The incubation was performed in SGF (0.15 M NaCl adjusted with 1M HCl to pH 2) at 37°C. Aliquots were taken at 0 and 120 min and analyzed by SDS-PAGE and RP-HPLC. The digestion was stopped by raising the pH to 7.4 by addition of 50 mM Na-Phosphate pH 7.4 and the samples were stored at −20°C. LTP samples were subjected to trypsin digestion (Roche Diagnostics GmbH, Germany) using an enzyme/substrate ratio of 1: 20 w/w. The incubation was performed in 1% ammonium bicarbonate, at 37°C. Aliquots were taken at 0 and 120 min and analyzed by SDS-PAGE and RP-HPLC. The digestion was stopped by boiling the sample for 2 min. The samples were then vacuum dried, washed three times with water to remove traces of ammonium bicarbonate, solubilized in PBS and stored at −20°C.

### Act d 10 primary structure determination and analysis

Amino acid sequencing of LTP N-terminal region was performed with an Applied Biosystems Procise 492 automatic sequencer (Applied Biosystems, Foster City, CA). Denaturation and alkylation of LTP sulfhydryl groups with 4-vinylpyridine was carried out as already described [21]. Denatured LTP was divided into two aliquots and subjected to proteolytic cleavage by either trypsin or Asp-N following manufacturer's instructions (Roche Diagnostics GmbH, Mannheim, Germany). Due to the large size, a peptide deriving from digestion with Asp-N was sub-digested with chymotrypsin (Roche Diagnostics GmbH). Separation of the peptides obtained by proteolytic cleavages and amino acid sequencing were performed as already described [21]. Protein sequence analyses were performed using available software on the ExPASy Proteomics Server (www.expasy.org).

### Biotinylation of kiwifruit LTPs

Purified nAct c 10 and nAct d 10 preparations (1 mg/ml, in 0.1 M sodium carbonate buffer pH 8.7) were incubated with 5 times molar excess of biotin N-hydroxysuccinimide ester (Sigma-Aldrich, Milano, Italy) dissolved in DMSO (Sigma-Aldrich). After 3.5 hours at room temperature, the excess reagents were removed by gel-filtration chromatography using a PD10 column (Amersham Biosciences, Uppsala, Sweden) equilibrated in PBS. The biotinylated preparations were then used for coupling with the streptavidin-CAP as described above.

### Protein solutions for skin test

Dialyzed protein extracts and purified LTP samples in deionized water were mixed with sterile glycerin in a 1∶1 ratio. The final protein concentration was 0.5 mg/ml. The LTP solutions were sterilized by membrane filtration through a 0.22*-*µm filter (Millex, Millipore, Bedford, MA, USA), in a sterile horizontal laminar flow hood.

### Data storing, processing and Statistics

All diagnostic and experimental data were stored into the InterAll *e*-record and underwent descriptive statistics including prevalence and median value for quantitative variables. Prevalence comparisons have been carried out using the χ^2^ test followed by pair-wise comparison using the Tukey post hoc test. Significances in inter-group value correlation comparisons were evaluated by the Spearman test. Specific IgE and ST results were expressed using median value, with interquartile ranges (5th percentile-95th percentile) (IQR). Serum IgE values were analyzed using the Kruskal-Wallis One-Way ANOVA by Ranks and the pair-wise comparison with a Mann-Whitney test with Bonferroni correction. Serum IgE percent value distribution obtained after the inhibition assay were compared using the Kruskal-Wallis One-Way ANOVA by Ranks, followed by the post hoc test Nemenyi-Damico-Wolfe-Dunn. The χ^2^ or Fisher's exact tests were applied to contingency tables and used when appropriate depending on the number of observations. All tests were used with two-sided options and significance level was set at a p value<0.05. Statistical analysis and graphical visualization of data have been performed using the R software (www.r-project.org) and Graphpad Prism (version 5.02, Graphpad Software Inc., La Jolla, CA, USA).

## Results

### Detection of LTP in kiwifruit pulp and seed protein extracts

Seed and pulp extracts of green and gold KF were prepared, fractionated by RP-HPLC and the separated protein components were manually collected and analyzed by N-terminal amino acid sequencing. The analysis of a peak eluted at a retention time very similar to that observed for nPru p 3 allowed the identification of the LTP, namely Act c 10 and Act d 10, in the seed extracts from the gold and the green KFs, respectively, rather than from the pulp (see below). Act c 10 was eluted as a single peak, whereas Act d 10 was eluted in three overlapping peaks suggesting the presence of some isoforms. Direct protein sequencing of the purified protein allowed the identification of two different isoforms of Act d 10 (see below). Possible additional isoforms were not identified probably because of the low yield. Conversely, the LTP peak was absent in the RP-HPLC profiles obtained for the pulp extracts of the two KF species.

### nAct c 10 and nAct d 10 purification

The recovery of the pure protein was about 0.4 mg per gram of green and gold KF seeds. The pulp was not used for LTP preparation. Protein concentration was estimated on the basis of the molar extinction coefficient at 280 nm (3480 M^−1^ cm^−1^). Purity of the protein preparations was checked by SDS-PAGE, RP- HPLC and N-terminal amino acid sequencing.

### Full primary structure elucidation of nAct d 10

**Figure 1:**
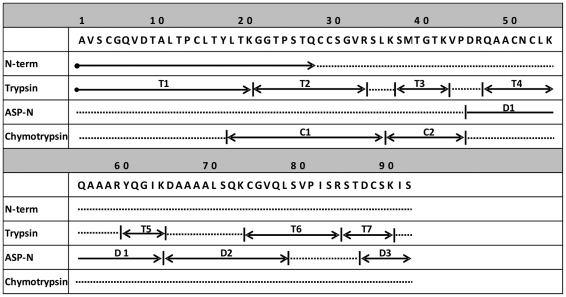
Act d 10 complete primary structure. Arrows indicate fragments obtained by enzymatic digestion with trypsin (T), Asp-N (D) and chymotrypsin (C). The amino acid sequence of the N-terminal region obtained by direct sequencing of the entire molecule is indicated by N-term. Peptides are numbered according to their order in the sequence.

The direct sequencing of the N-terminal region of native nAct d 10 produced 17 identifiable amino acid residues, AVSCGQVDTALTPCLTY (Figure 1). A small fraction of the molecules contained the amino acid threonine (T) rather than alanine (A) as first residue. The N-terminal sequence of nAct c 10, comprising 17 amino acid residues, was identical to that of nAct d 10 (AVSCGQVDTALTPCLTY), and no heterogeneity at the first position was observed. The complete amino acid sequence of nAct d 10 was elucidated by automated sequencing of peptides resulting from the enzymatic digestion of denatured and S-pyridilethylated protein. Most of the primary structure was obtained by aligning the amino acid sequence of peptides from trypsin and Asp-N digestions, whereas the regions corresponding to the residues 18–35 and 36–42 were obtained by sequencing peptides from chymotrypsin digestion. Figure 1 shows only the peptides necessary to elucidate the complete amino acid sequence. nAct d 10 comprises 92 amino acids producing a molecular mass of 9,458 Da for the most abundant isoform, having alanine at the N-terminus. The 100% sequence identity in the N-terminal region, together with the observation that nAct d 10 and nAct c 10 have the same chromatographic behavior and very similar molecular masses as estimated by mass spectrometry analyses (see below), suggested a very high structural similarity between the two proteins. Therefore, only the full nAct d 10 primary structure was elucidated. The full amino acid sequence of the nAct d 10 isoforms having alanine or threonine as N-terminal residue, and the partial N-terminal sequence of nAct c 10, have been registered in the UniProt Knowledgebase with the accession numbers P85205, P85206, and P85204, respectively. Act c 10 and Act d 10 are allergen names approved by the WHO-IUIS Allergen Nomenclature Subcommittee (www.allergen.org), and encoded 5735 and 5737, respectively, in the Allergome database (www.allergome.org).

### Estimation of the molecular mass by mass spectrometry

The analyses by MALDI-TOF mass spectrometry of purified nAct d 10 provided two values, 9.464 (±20) and 9.484 (±20) kDa, in good agreement with the mass values deduced from the amino acid sequence of the two isoforms having alanine (9.458 kDa) or treonine (9.488 kDa) as N-terminal residues, respectively. A molecular mass of 9.460 (±20) kDa was obtained for Act c 10 by mass spectrometry.

### Evaluation of the resistance to proteolysis

The analysis by SDS-PAGE and RP-HPLC of the LTP samples subjected to digestion in SGF showed that nAct c 10 and nAct d 10, similarly to nPru p 3, are resistant to gastric digestion. Moreover, in line with the results reported by Cavatorta et al. [22], nPru p 3 was partially digested by trypsin, whereas nAct d 10 and nAct c 10 appeared to be resistant to the same digestion (data not shown).

### Sequence identity and alignments among LTPs under study

**Figure 2:**
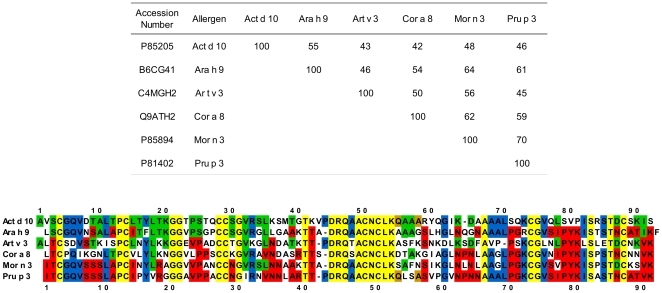
Amino acid identity table and sequence alignments of Act d 10, Ara h 9, Art v 3, Cor a 8, Mor n 3, and Pru p 3. Panel A: Table of amino acid identity reported as paired percent values; Panel B: LTP sequence alignments; Act d 10 and Pru p 3 sequence numbering are indicated at the top and at the bottom of the alignments, respectively. Background colors identify identical amino acids as follows: Yellow: identical amino acids in all six sequences; Blue: identical amino acids in Act d 10 and Pru p 3 and some other LTP sequences; Green: amino acids of Act d 10 substituted in Pru p 3, but conserved in at least one of the other sequences; Red: amino acids of Pru p 3 substituted in Act d 10, but conserved in at least one of the other sequences; Brown: identical amino acids only in Act d 10 and Pru p 3 sequences; White: no amino acid identities with Act d 10 and Pru p 3 sequences.

Homology search in Uniprot protein database carried out using the BLAST algorithm (www.expasy.org) showed the sequence identity between Act d 10 and other already known allergenic LTPs to be not very high, ranging between 55% with Ara h 9 (isoform Ara h 9.0201) and 35% with Par j 2. Identities among the full length amino acid sequence of the six allergenic LTPs under study (Act d 10, Ara h 9, Art v 3, Cor a 8, Mor n 3 and Pru p 3) are comprised in the 42–70% range (Figure 2, panel A). The sequence identity values between Act d 10 and other allergenic LTPs, such as Api g 2 (celery stalk), Cit s 3 (orange), Fra a 3 (strawberry), Lac s 1 (lettuce), Len c 3 (lentil), Lyc e 3 (tomato), Mal d 3 (apple), Ory s 14 (rice), Pla or 3 (plane tree pollen), Pru du 3 (almond), Pyr c 3 (pear), Sin a 3 (mustard), Tri a 14 (wheat), Vit v 1 (grape), Zea m 14 (maize), are comprised in the narrow range of 40–55% (data not shown). The alignment of the amino acid sequences of the LTPs analyzed in the present study (Figure 2, panel B) underlines that the best conserved region is a stretch of 10 contiguous residues (positions 45–54, Act d 10 numbering, Figure 2, panel B). Twenty-four amino acids (26%) (yellow background) are conserved in all the six aligned sequences, including the eight cysteine residues. Act d 10 shares 18 additional residues with Pru p 3: two of them (brown background) are present only in Act d 10 and Pru p 3, whereas the remaining 16 shared residues (blue background) can be found also in other aligned sequences but not all of them. Twenty-one residues (23%) of Act d 10 are substituted in Pru p 3, whereas they are conserved in at least one of the aligned sequences (green background). A higher number of residues, namely 35 amino acids of Pru p 3 (38%), are substituted in Act d 10, but they are conserved in at least 1 of the other sequences (red background). Act d 10 and Pru p 3 do not share identical residues with any of the other aligned sequences in 27 and 14 sequence positions, respectively (white background).

### Defining the first set of kiwifruit allergic patients being sensitized to kiwifruit LTPs

Table 1 reports selected patients' diagnostic profiles. All patients reported a reliable clinical history of reactions when eating KF. The IgE screening performed using the ISAC test revealed none of the subjects to be sensitized by other KF allergens, namely nAct d 2, nAct d 5, nAct d 6, nAct d 7, nAct d 11, but one recorded positive to Act d 1, whereas all were recorded IgE positive to nPru p 3, the homologous peach LTP (Table 1, panel A). Patients were then tested with a commercial KF extract both by ST and CAP. Results were sometime weak, doubtful or even negative (Table 1, panel A). All patients underwent DBPCFC, all stopped because subjective and objective allergic symptoms appeared as reported in Table 1, panel A. Thus this selected group of KF allergic patients underwent ST with the purified KF LTPs and IgE detection by mean of the biotinylated KF LTP coupled with the streptavidin-CAP. All ST and CAP testing were scored positive for the two KF LTPs (Table 1, panel B), whereas they were negative in 10 allergic subjects used as controls. The same patients were finally tested IgE positive for the two KF LTP preparations, along with the in-house nPru p 3 one, on the ISAC Exp96 (Table 1, panel B). The two proteins where thus considered clinically relevant allergens, and the ISAC Exp96 a suitable tool for further investigations.

**Table 1:** Diagnostic profiles of seven selected kiwifruit allergic patients used for the initial characterization of kiwifruit LTPs.

A.											
Subjects	InterAll APC Code	Age	Gender	Symptoms°	Total IgE	Skin test*		CAP IgE**	DBPCFC°	ISAC 103 IgE**	
						Commercial extract	Prick-Prick	Commercial extract		Act d 1	Pru p 3
1	ITROMIDI28837	26	M	URT	1254	18.00	Neg	Neg	URT	Neg	1.07
2	ITROMIDI21272	40	M	URT	790	Neg	Neg	4.10	URT	Neg	1.01
3	ITROMIDI24698	37	F	OAS	11	43.69	89.05	1.24	URT	Neg	3.61
4	ITROMIDI24232	31	F	GI - URT	65	Neg	29.39	1.02	GI - URT	Neg	1.89
5	ITROMIDI28626	16	M	GI	450	Neg	60.06	3.40	URT	4.34	9.35
6	ITROMIDI1558	28	M	GI - URT	211	56.00	109.11	0.84	GI - URT	Neg	2.70
7	ITROMIDI5272	27	F	OAS	2500	17.34	23.76	7.00	URT	Neg	5.46

B.											
Subjects	InterAll APC Code	Age	Gender	Skin test*			CAP IgE**		ISAC Exp96 IgE**		
				Act d 10	Act c 10	Pru p 3	Act d 10	Act c 10	Act d 10	Act c 10	Pru p 3°°
1	ITROMIDI28837	26	M	15.00	100.40	78.00	0.74	0.93	0.70	0.43	2.59
2	ITROMIDI21272	40	M	27.00	36.00	12.00	25.90	65.90	9.91	10.41	6.62
3	ITROMIDI24698	37	F	90.53	25.51	98.30	4.70	4.13	1.58	1.50	2.85
4	ITROMIDI24232	31	F	67.82	40.16	57.68	8.68	12.30	12.64	13.99	5.14
5	ITROMIDI28626	16	M	74.98	69.50	129.00	26.8	30.20	4.12	6.29	17.02
6	ITROMIDI1558	28	M	97.07	98.69	98.00	1.02	1.20	0.49	0.60	4.37
7	ITROMIDI5272	27	F	95.50	85.40	72.57	88.4	100.00	22.84	15.65	21.51

Panel A: Tests leading to kiwifruit LTP suspected reactivity are reported.

APC: Allergome Personal Code used in the InterAll e-record.

° Double Blind Placebo Controlled Food Challenge; GI: gastro-intestinal tract symptoms including vomiting and abdominal pain; OAS: oral allergy syndrome; URT: Urticaria.

*Results of skin testing performed with green kiwifruit are expressed as mm^2^.

**ISAC and CAP IgE results are expressed as kU/l.

Panel B: *In vivo* and *in vitro* results of kiwifruit LTP testing are reported.

APC: Allergome Personal Code used in the InterAll *e*-record.

*Results of skin testing are expressed in mm^2^.

**ISAC and CAP IgE results are expressed as kU/l. A streptavidin-CAP has been used for kiwifruit LTP IgE detection.

°° In-house Pru p 3 preparation used throughout the study was immobilized on ISAC Exp96.

**Figure 3:**
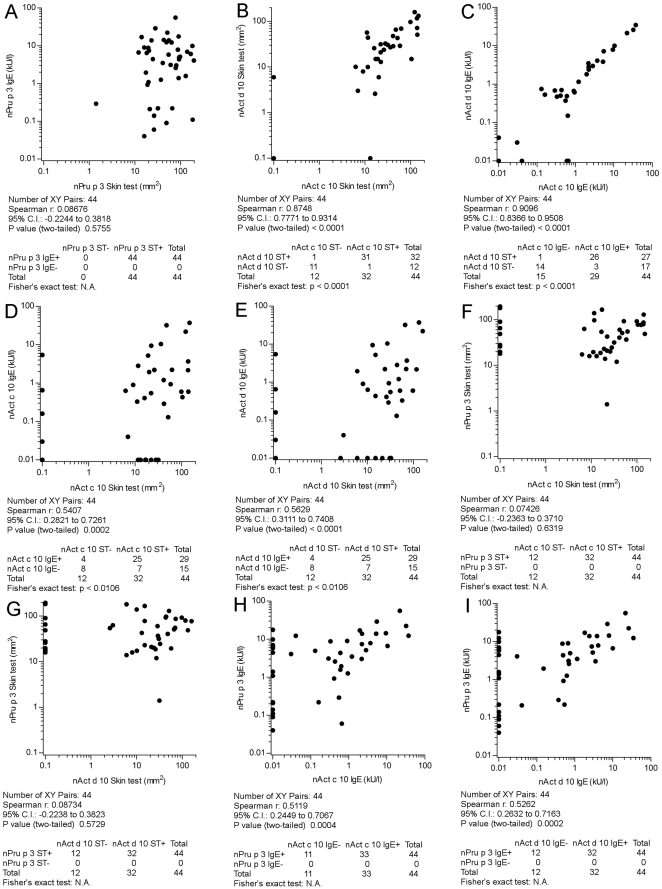
Comparative skin testing and IgE detection using nAct c 10, nAct d 10, and nPru p 3 in 44 peach clinically allergic subjects tested positive for nPru p 3 on ISAC 103. Panels A to I report correlation and concordance results for each paired allergen preparation and test as in the graphs. The Spearman r correlation coefficient and the Fisher's exact test have been used for statistical evaluations. Statistics are reported below each graph. IgE results have been obtained using ISAC microarray Exp96 and reported as kU/l; skin test have been obtained by measuring wheal areas and reported as mm^2^_._ For graphical visualization needs on log scales, zero values for the skin test have been set at 0.1 mm^2^, and at 0.01 kU/l for ISAC values.

To confirm the findings of the previous set of tests and to start a comparative evaluation of LTPs from different sources, a preliminary extended *in vivo* and *in vitro* testing has been performed by ST and by detecting IgE on ISAC Exp96, using identical nAct c 10, nAct d 10, and nPru p 3 preparations in the two tests. Forty four patients complaining about symptoms on eating peach, enrolled on the basis of a positive DBPCFC or a reliable history of recent anaphylaxis and tested positive for nPru p 3 on the routine ISAC 103, were evaluated. Table 2 reports data obtained by ST and IgE detection in this selected cohort of patients. All 44 subjects had a positive ST to nPru p 3 (median wheal area 48.83 mm^2^, range 1.4–195) confirming the positive IgE result on ISAC 103 used for selection, and replicated on ISAC Exp96 (median 4.68 kU/l, range 0.1–55.6), without statistically significant differences when compared to ISAC 103 results. A slightly not statistically significant different skin reactivity was recorded when considering nAct c 10 (median wheal area 18.0 mm^2^, range 6.2–148.6) and nAct d 10 (median wheal area 15.0 mm^2^, range 2.6–159) (Table 2). To set the relationship between ST and IgE detection, nPru p 3 results obtained in the two tests were compared; no correlation was found between ST areas and IgE values (Figure 3, panel A). When the ST was performed using the two KF LTPs 31 subjects reacted to both allergen preparations, whereas positive ST was recorded to one and not to the other in one subject each (Figure 3, panel B). Similarly, IgE results detected for the two molecules were compared without significant differences (Figure 3, panel C). Differing from nPru p 3 ST *versus* IgE data analysis, a non-overlapping reactivity, anyhow not statistically different, has been found when considering nAct c 10 and nAct d 10 ST *versus* IgE positive subjects, but the correlation between the ST induced wheal areas and the IgE values was statistically significant, though the Spearman r value was just 0.54 and 0.56 for the two LTPs, respectively (Figure 3, panel D, E). Finally, no statistically significant correlations were found when ST and IgE results were compared between nAct c 10 and nAct d 10 *versus* nPru p 3 (Figure 3, panel F, G, H, I).

**Table 2:** Skin test and IgE data on 44 peach allergic subjects.

	Skin Test*			ISAC Exp96 IgE°		
	nPru p 3	nAct c 10	nAct d 10	nPru p 3	nAct c 10	nAct d 10
Tested	44	44	44	44	44	44
Positive	44	32	32	44	29	27
%	100	72.7	72.7	100	65.9	61.4
Median	48.83	18.00	15.00	4.68	0.42	0.48
Min	1.40	6.20	2.60	0.10	0.10	0.10
Max	195.00	148.65	159.00	55.60	37.12	34.57

*Skin test reactivity is reported as wheal area (mm^2^).

° ISAC Exp IgE values are reported as kU/l.

Overall the findings reported above preliminary marked immunological differences between the LTPs from peach and KFs, thus suggesting that the shared LTP reactivity recorded in the first set of patients and leading to the identification of KF LTPs could not be extended to all nPru p 3 positive subjects.

### Defining the magnitude of kiwifruit LTP reactivity by comparative skin testing and IgE detection toward other LTPs

As from the previous section findings, showing heterogeneity between LTPs from two allergenic sources in that small study group, a larger study was set using the routine testing approach by the ISAC microarray, in order to define any possible subsets related to different LTP IgE recognition. A comparative evaluation of seven LTPs was undertaken using the ISAC 103 and ISAC Exp96 microarrays in parallel.

**Figure 4:**
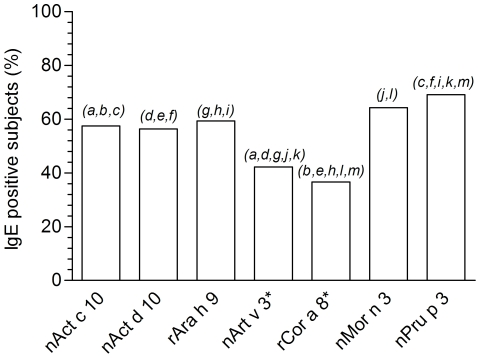
IgE prevalence for nAct c 10, nAct d 10, rAra h 9, nArt v 3, rCor a 8, nMor n 3, nPru p 3 on 431 sera. Prevalence has been calculated on 431 patients having at least one positive IgE test for one of the LTP under study. Asterisk marks LTPs tested on ISAC 103; remaining have been tested on ISAC Exp96. Statistical comparative evaluation has been performed using the χ^2^ test to evaluate the overall differences among all seven values. Pair-wise comparison has been performed using the Tukey post hoc test. “p” values, for paired letters in italics on top of the two corresponding bars, were as follows: *a*: p = 0.00015; *b*, *e*, *g*, *h*, *j*, *k*, *l*, *m*: p<0.0001; *c*: p = 0.0076; *d*: p = 0.00067; *f*: p = 0.0021; *i*: p = 0.045.

Unless the original two population were defined on the basis of being nPru p 3 IgE positive or negative as tested on ISAC 103, additional nPru p 3 positive subjects were found in the control population using the ISAC Exp96 microarray bearing a different nPru p 3 preparation, thus raising the number of nPru p 3 positive subjects from 259 to 296. The same control population revealed a number of subjects being nPru p 3 IgE negative but positive to at least one of the other LTPs, increasing the overall number of subjects being positive to at least one of the LTPs from 296 to 431. nAct c 10, nAct d 10, rAra h 9, nArt v 3, rCor a 8, and nMor n 3 were positive when nPru p 3 was negative in 48, 41, 17, 56, 43, and 17 cases, respectively. The number of subjects positive to one LTP as listed above and negative to all others was relatively low, being 5, 0, 9, 25, 10, 9, 17. These prevalence were statistically different only when considering nArt v 3 values *versus* KF LTPs, peanut and mulberry ones. Absence of nAct d 10 exclusively IgE positive subjects statistically differed to all the remaining LTPs rather than nAct c 10 (data not shown). Raw prevalence are reported in Figure 4, calculated on the subset of 431 patients tested positive to at least one of the seven LTPs. An overall significant p<0.0001 of the χ^2^ test has been obtained taking together the seven prevalence, whereas not all prevalence were statistically different when compared each other. For instance, nPru p 3 was anyway the most prevalent sensitization with no difference only when compared to nMor n 3. IgE sensitization to nAct c 10 and nAct d 10 had statistically significant higher prevalence than the mugwort and the hazelnut LTPs, but less prevalent than nMor n 3 and nPru p 3, though proven by statistics just for nPru p 3. The less prevalent IgE reactivity have been recorded for the mugwort and the hazelnut LTPs being statistically different from all other LTPs. Detailed statistics are reported in Figure 4.

**Figure 5:**
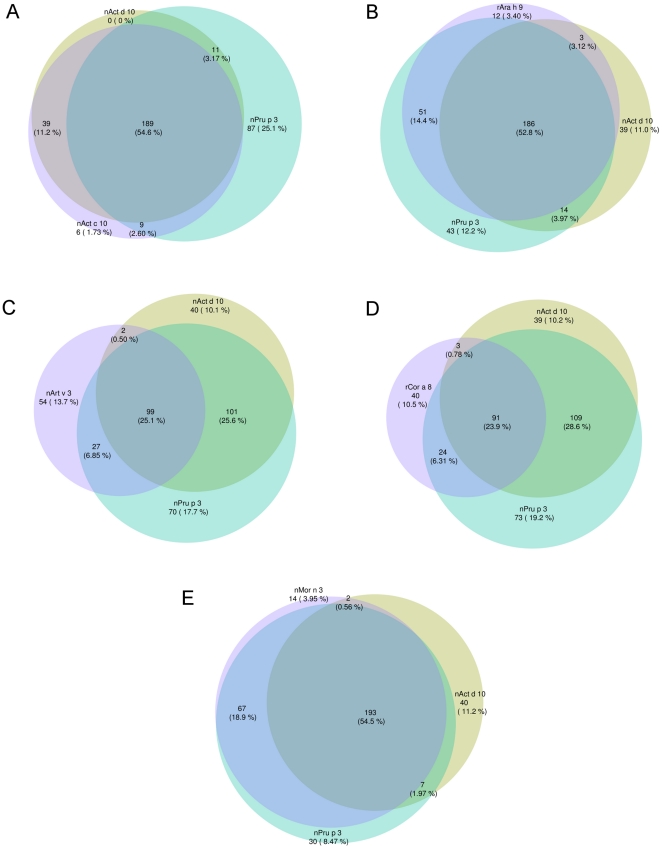
Venn diagram representation of positive IgE results for selected LTPs. LTP specific IgE have been determined using ISAC Exp96, excepting for nArt v 3 and rCor a 8where ISAC 103 has been used. Due to the highly similar behavior of the two kiwifruit LTPs, panel from B to E show how the other four LTPs behave compared to nAct d 10 and nPru p 3. Absolute and relative IgE prevalence are given for each combination on graphs as follows: Panel A: nAct c 10, nAct d 10, and nPru p 3; Panel B: nAct d 10, nPru p 3, rAra h 9; Panel C: nAct d 10, nPru p 3, nArt v 3; Panel D: nAct d 10, nPru p 3, rCor a 8; Panel E: nAct d 10, nPru p 3, nMor n 3.

To visualize exclusive sensitization to one LTP compared to others, a series of Venn diagrams showing all possible logical relations between our finite collections of IgE values obtained by the two microarray testing, have been generated and reported in Figure 5. The first Venn diagram as reported in Figure 5, panel A, shows almost overlapping results between the two KF LTPs, and non-overlapping results considering nAct c 10 and nAct d 10 opposed to nPru p 3. Due to the highly similar behavior of the two KF LTPs, the following Figure 5 panels show how each of the other four LTPs under study behaves compared to nAct d 10 and nPru p 3. Venn diagrams change depending on individual LTP features, further showing heterogeneity of the members of this group of molecules. An index of the different reciprocal behavior of the LTPs is the changing number of isolated positivity to the three LTPs used in each graph depending on the third LTP used.

**Figure 6:**
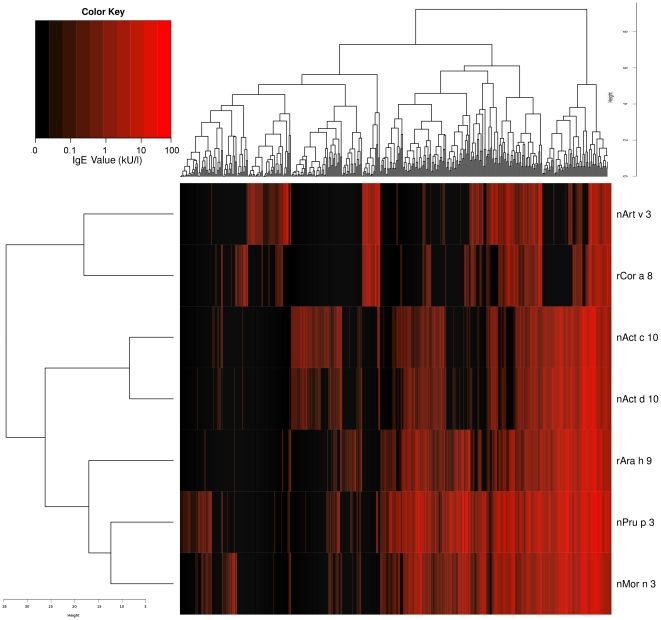
Unsupervised two-way hierarchical clustering analysis of 431 subjects tested for IgE on the seven LTPs. Logarithmic IgE value distribution has been used to generate the heat map. Subjects had at least one IgE-positive result to one LTP under study. LTP are reported on the y-axis, subjects on the x-axis with their respective relative distribution scales. Black to dark red scale corresponds to IgE values from negative to strongly positive. Color key legend gives an approx idea of visualized IgE values.

A cluster analysis performed using all subjects having IgE positive results to at least one of the seven LTPs generated the heat map shown in Figure 6. The overall picture shows the clustering heterogeneity of both allergens and patients. LTP IgE recognition by the sensitized subjects is well depicted for allergens by the presence of the first two clusters segregating nArt v 3 and rCor a 8 reactivity apart from the other LTPs. A second dichotomy is observed, separating the two KF LTPs from the remaining, also representing, as expected, the two molecules to be the closest ones. The remaining three LTPs form an additional cluster further divided in two. Subject clustering is dispersed in a great number of clusters, much reflecting the individual heterogeneity of LTP IgE recognition.

**Figure 7:**
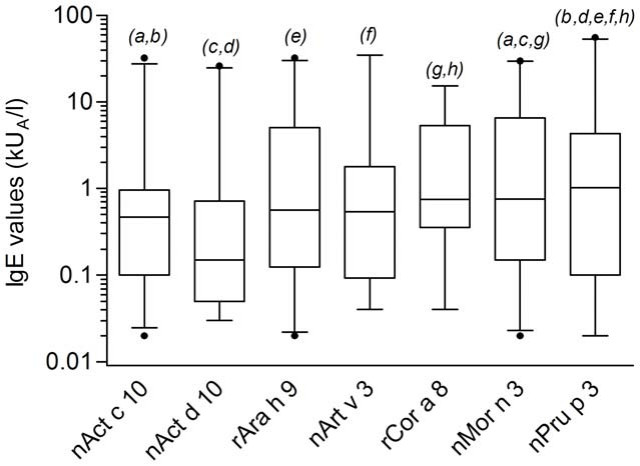
IgE values box-plot representation considering median values and 5–95 percentile distributions. IgE value distribution is plotted for positive values. Kruskal-Wallis One-Way ANOVA by Ranks test gave a statistically significant p value (<0.0001). The pair-wise comparison with a Mann-Whitney test with Bonferroni correction applied to LTP IgE results gave p values, considering paired letters in italics on top of the two corresponding bars, as follows,: *a*: p = 0.00218; *b*, *d*, *h*: p<0.0001; *c*: p = 0.00014; *e*: p = 0.00093; *f*: p = 0.00079; *g*: p = 0.03402.

Beside the evaluation of positive/negative results reported above and clearly showing non-overlapping behavior among LTPs, we sought to verify whether detected IgE value distributions were different comparing the seven LTPs under study. A Kurskal-Wallis test gave statistically significant results (p<0.0001), and, when comparing the paired groups using the Mann-Whitney test several statistically different findings were recorded at different p values (Figure 7). Differing from prevalence results reported in Figure 4, nAct c 10 and nAct d 10 showed the lowest median values, having those within the 5–95 percentiles distributed in a quite narrow range (Figure 7). Both their distributions did not differ significantly from rAra h 9, nArt v 3, and rCor a 8 values, whereas statistically significant differences were obtained when comparison was carried out toward nMor n 3 and nPru p 3 IgE value distributions. rAra h 9 and nArt v 3 behaved almost the same differing only from nPru p 3. Overall evaluating the distributions of IgE values further described the behavioral heterogeneity within LTPs as allergens.

**Figure 8:**
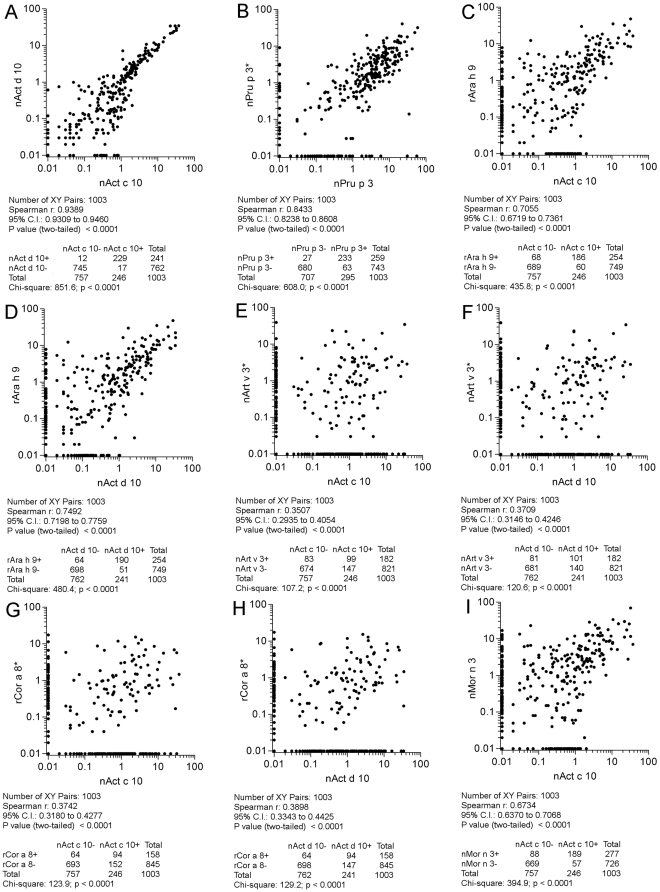
IgE value correlations for paired LTPs. All 1,003 subjects have been plotted in each graph. Flags A, B, C, D, E, F, G, H, I in figure 8 indicate them as parts of the results shown also in figures 9, and 10. Consecutive letters have been used on purpose through the three figures. The * marks IgE detection performed on ISAC 103, remaining IgE determinations have been obtained by ISAC Exp96. For graphical visualization needs on log scales, zero values for ISAC testing have been set to 0.01 kU/l. The Spearman r correlation coefficient has been calculated and the χ^2^ test has been used for statistical purposes. Statistics are reported below each graph.

**Figure 9:**
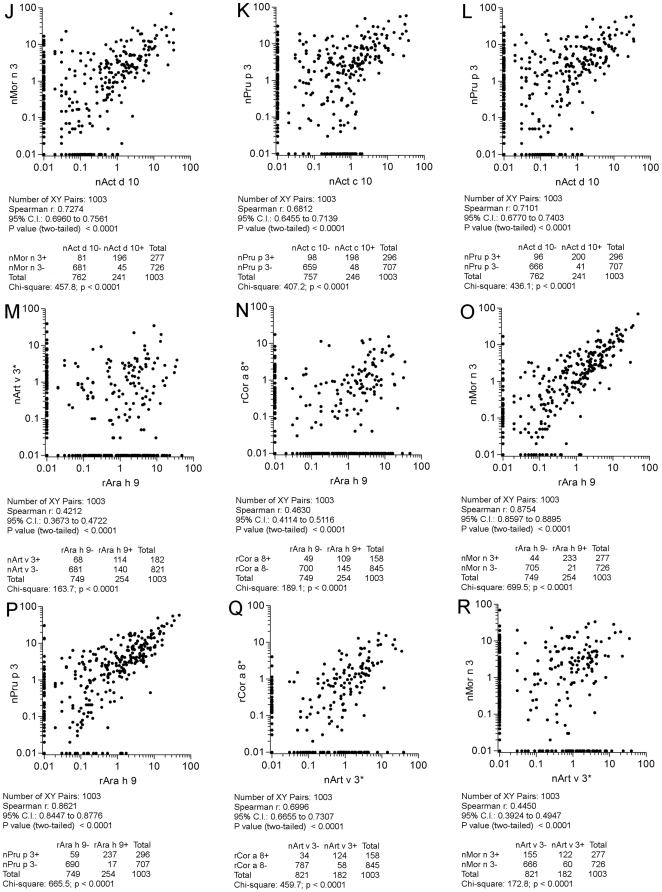
IgE value correlations for paired LTPs. All 1,003 subjects have been plotted in each graph. Flags J, K, L, M, N, O, P, Q, R in figure 9 indicate them as parts of the results shown also in figures 8, and 10. Consecutive letters have been used on purpose through the three figures. The * marks IgE detection performed on ISAC 103, remaining IgE determinations have been obtained by ISAC Exp96. For graphical visualization needs on log scales, zero values for ISAC testing have been set to 0.01 kU/l. The Spearman r correlation coefficient has been calculated and the χ^2^ test has been used for statistical purposes. Statistics are reported below each graph.

**Figure 10:**
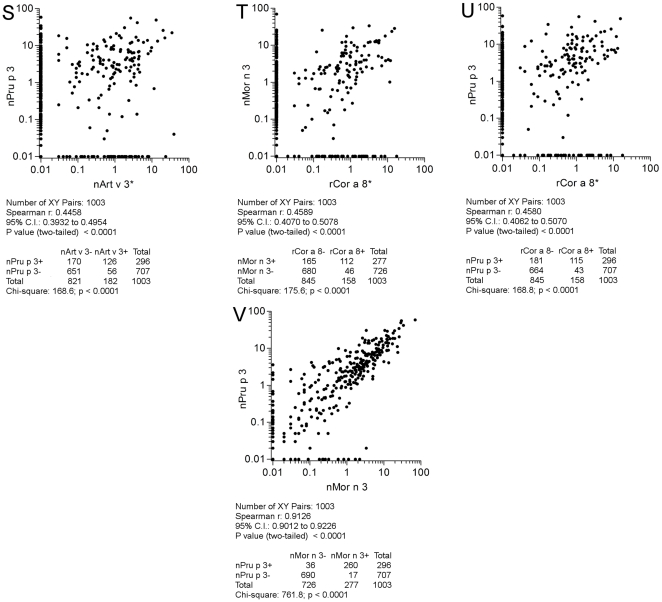
IgE value correlations for paired LTPs. All 1,003 subjects have been plotted in each graph. Flags S, T, U, V in figure 10 indicate them as parts of the results shown also in figures 8, and 9. Consecutive letters have been used on purpose through the three figures. The * marks IgE detection performed on ISAC 103, remaining IgE determinations have been obtained by ISAC Exp96. For graphical visualization needs on log scales, zero values for ISAC testing have been set to 0.01 kU/l. The Spearman r correlation coefficient has been calculated and the χ^2^ test has been used for statistical purposes. Statistics are reported below each graph.

All the 1,003 paired results were then plotted considering paired allergens as reported in Figure 8, panels A-I; Figure 9, panels J-R; Figure 10, panels S-V. All comparisons were highly statistically significant (p<0.0001). As reference, both for statistical findings and graphical representations, the nAct c 10 *versus* nAct d 10 correlation was taken, having the highest Spearman r (0.94) and χ^2^ (851.6) values (Figure 8, panel A). Twenty-nine discrepant results out of 258 positive ones (11.2%) for the two KF LTPs were recorded, being the lowest value among the others. Discrepant results are clearly shown along the X and Y axis and reported in the table underneath each graph. Taking advantage of the availability of two different nPru p 3 preparations on the two microarrays in use, we plotted and evaluated their IgE results, giving the Spearman r = 0.84 and a χ^2^ = 608 values as shown in Figure 8, panel B. Unless the statistics were very good, a higher number of discrepant results (n = 90; 27.9%) were recorded compared to the above reported results obtained with two KF LTPs. A very good correlation was recorded again just when comparing nMor n 3 and nPru p 3 (r = 0.91 and a χ^2^ = 761.8) (Figure 10, panel V), showing also the highest number of concordant positive results (n = 260), whereas all other correlations showed a quite broad range of values, being the lowest r (0.35) and χ^2^ (107.2) when comparing nAct c 10 and nArt v 3 (Figure 8, panel E), and the highest (0.88; 699.5) when rAra h 9 and nMor n 3 IgE results were matched (Figure 9, panel O). In many cases IgE plotted points showed no linearity at all, being scattered in a broad area (Figure 8, panels E, F, G, H; Figure 9, panels M, R; Figure 10, panel S). In other cases a certain linearity was still present for part of the subjects, but part of the values were anyhow plotted above or below the theoretical central line (Figure 8, panels C, D, I; Figure 9, panels J, K, L, N, O, P, Q; Figure 10, panels T, U). The highest number of discrepant IgE results were recorded for the paired nArt v 3/nPru p 3 results (n = 226; 64.2%) (Figure 10, panel S), whereas those recorded for rAra h 9/nMor n 3 (n = 65; 21.8%) were very close to the best ones reported above (Figure 9, panel O). Overall evaluating IgE correlations among allergens under study shed further light on LTP immunological heterogeneity.

### IgE inhibition experiments

**Figure 11:**
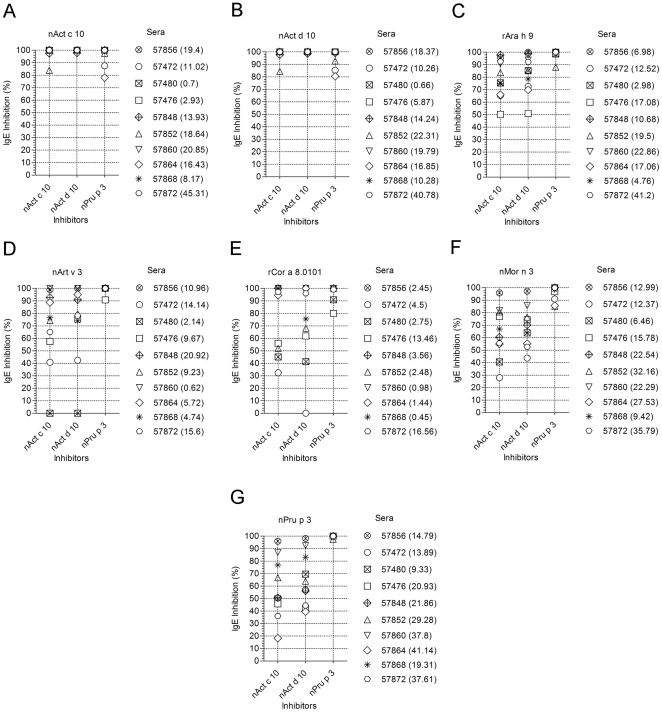
Single Point Highest Inhibition Achievable assay (SPHIAa) for LTP IgE inhibition. IgE values on graphs are reported as percent inhibition. IgE values for each serum in each graph representation are given in brackets. The three inhibitors are indicated in the X axis, whereas on top of each graph the LTP immobilized on the microarray whose IgE binding has been inhibited is reported. Statistical evaluations have been applied to the three series using the Kruskal-Wallis One-Way ANOVA by Ranks , followed by the *post hoc* test of Nemenyi-Damico-Wolfe-Dunn comparing paired series. Statistics are as follows: **Panel A - nAct c 10**: nAct c 10 Median = 100%; Range 83.91–100%; nAct d 10 Median = 100%; Range 97.92–100%; nPru p 3 Median = 100%; Range 78.03–100%; Kruskal-Wallis = n.s.s.; **Panel B - nAct d 10**: nAct c 10 Median = 100%; Range 84.27–100%; nAct d 10 Median = 100%; Range 98.67–100%; nPru p 3 Median = 100%; Range 80.42–100%; Kruskal-Wallis = n.s.s.; **Panel C - rAra h 9**: nAct c 10 Median = 79.62%; Range 50.18– 97.85%; nAct d 10 Median = 85.39%; Range 51.00–100%; nPru p 3 Median = 100%; Range 88.05–100%; Kruskal-Wallis p = 0.00061; nAct c 10 *vs* nAct d 10 = n.n.s.; nAct c 10 *vs* nPru p 3 p = 0.00019; nAct c 10 *vs* nPru p 3 p = 0.0055; **Panel D - nArt v 3**: nAct c 10 Median = 75.45%; Range 0–100%; nAct d 10 Median = 79.04%; Range 0–100%; nPru p 3 Median = 100%; Range 90.80–100%; Kruskal-Wallis p = 0.0012; nAct c 10 *vs* nAct d 10 = n.n.s.; nAct c 10 *vs* nPru p 3 p = 0.00078; nAct c 10 *vs* nPru p 3 p = 0.0083; **Panel E - rCor a 8**: nAct c 10 Median = 95.83%; Range 32.44–100%; nAct d 10 Median = 85.79%; Range 0–100%; nPru p 3 Median = 100%; Range 80.01–100%; Kruskal-Wallis p = n.n.s.; **Panel F - nMor n 3**: nAct c 10 Median = 63.71%; Range 27.97–95.84%; nAct d 10 Median = 67.06%; Range 43.65–97.31%; nPru p 3 Median = 98.99%; Range 85.11–100%; Kruskal-Wallis p = 0.00031; nAct c 10 vs nAct d 10 = n.n.s.; nAct c 10 vs nPru p 3 p = 0.00035; nAct c 10 vs nPru p 3 p = 0.0011; **Panel G - nPru p 3**: nAct c 10 Median = 50.21%; Range 18.25–95.94%; nAct d 10 Median = 60.55%; Range 39.55–98.11%; nPru p 3 Median = 100%; Range 97.44–100%; Kruskal-Wallis p<0.0001; nAct c 10 vs nAct d 10 = n.n.s.; nAct c 10 vs nPru p 3 p<0.0001; nAct c 10 vs nPru p 3 p = 0.0003.

To define immunological relationships in terms of IgE binding among LTPs, the SPHIAa assay was run using nAct c 10, nAct d 10, and nPru p 3 as inhibitor on the seven LTPs. The assay was performed using ten individual sera selected for being IgE positive to the three inhibitors as shown in Figure 11, where IgE values for any given inhibited LTP are given for each of the ten sera. All the positive allergens other than LTPs used for control purposes for each of the samples gave no inhibition values (data not shown). Full autologous IgE inhibitions on the two KF LTPs were achieved, as well as with the homologous nPru p 3 preparation (Figure 11, panel A and B). As shown in Figure 11, panel C to F, nPru p 3 fully inhibited all the other LTPs. The two KF LTPs showed a serum/allergen dependent behavior, having IgE inhibition values as follows: greater than 50% for all samples (rAra h 9, Figure 11, panel C); spread between 0 and 100% (nArt v 3, Figure 11, panel D); separated in two subgroups, one achieving 100% the other staying below 70% (rCor a 8, Figure 11, Panel E); hardly reaching 100%, and spread in a wide range, with the lowest value at 29% (nMor n 3, Figure 11, panel F); not reaching 100%, spread in a wider range, with the lowest value at 19% (nPru p 3, Figure 11, panel F). All statistics are given in Figure 11 legend. As for some of the other experiments reported above, minor differences between the two KF LTPs were recorded also in the inhibition test. Overall, also IgE inhibition experiments confirmed the immunochemical differences between the two new KF LTPs and the peach one and the different behavior in terms of epitope distribution, recognition and inhibition by the three inhibitors.

### Analysis of the LTP distribution within the kiwifruit tissues by biochemical, immunological and clinical methods

To verify whether the heterogeneous immunological behavior between the two KF LTPs and the peach LTP could lead to define a subset of patients who are also not clinically reactive to all LTPs, we first performed a series of experiments to define the LTP distribution within the KF tissues, namely the pulp and the seeds, based on biochemical, immunochemical and clinical methods.

**Figure 12:**
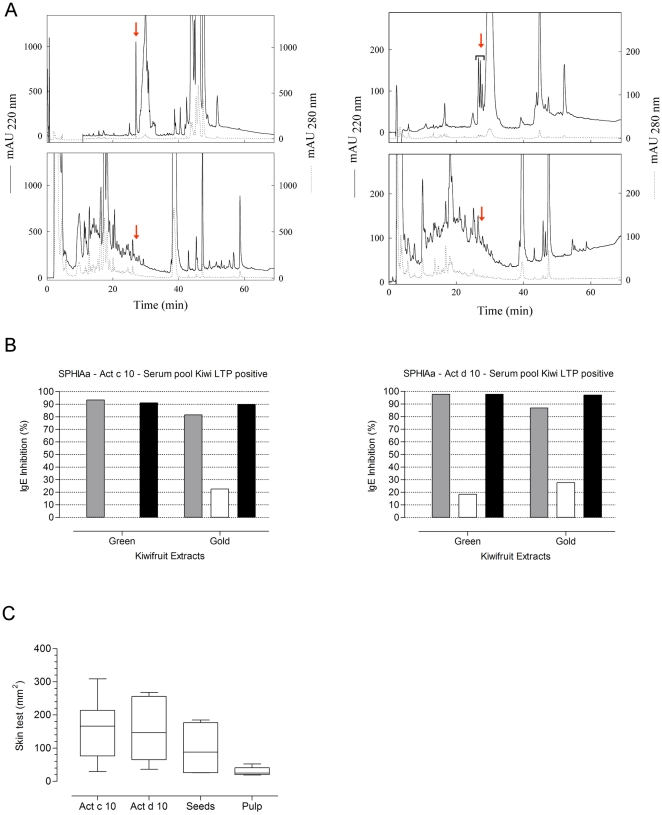
Biochemical, immunochemical, and clinical evaluation of LTP distribution in kiwifruit tissues. Panel A, Left: RP-HPLC profiles of the pulp (upper part) and seed (lower part) protein extracts of gold kiwifruit tissues. The amount of loaded proteins was 1 mg. The arrow indicates the elution time of Act c 10. Panel A, Right: RP-HPLC profiles of the pulp (upper) and seed (lower) protein extracts of green kiwifruit tissues. The amount of loaded proteins was 0.3 mg. The arrow indicates the elution time of Act d 10. Panel B: Single Point Highest Inhibition Achievable assay (SPHIAa) using pulp and seed extract preparations from green and gold kiwifruits. Grey bars: total kiwifruit extracts; White bars: bars: kiwifruit pulp extracts; Black bars: kiwifruit seed extracts. Panel C: Skin test using nAct c 10, nAct d 10, and green kiwifruit seed and pulp preparations. Skin test wheal areas have been recorded and expressed in mm^2^. The Mann-Whitney test applied to paired test result distributions gave p values as follows: Act c 10 *vs* Act d 10 p = n.n.s.; Act d 10 *vs* Seeds p = n.n.s.; Act d 10 *v*s Pulp p<0.004; Seeds *vs* Pulp p<0.017.

As reported in the first paragraph of the Result section, using biochemical methods, seed and pulp extracts were fractionated by RP-HPLC (Figure 12, panel A) and the separated protein components were analyzed by N-terminal amino acid sequencing. The analysis of a peak eluted at a retention time very similar to that observed for nPru p 3 allowed the identification of nAct c 10 and nAct d 10 in the seed extracts from the gold (Figure 12 A, left) and the green KFs (Figure 12 A, right), respectively (Figure 12, panel A, upper parts), whereas that peak was lacking in the RP-HPLC profiles obtained for the pulp extracts of the two KF species (Figure 12, panel A, lower parts). Nevertheless, the fractions of pulp extracts eluted at the retention time of nAct d 10 and nAct c 10 were collected and analyzed by N-terminal amino acid sequencing. The results obtained confirmed the absence of detectable amounts of nAct d 10 and nAct c 10 in the pulp extracts. As the biochemical findings reported above seemed to be quite conclusive, to increase the confidence with the negative data we approached the issue of LTP distribution in KF tissues using the SPHIAa as well. The inhibition was performed using the same pulp and seed preparations from the green and gold KFs, along with whole KF extracts. A pool of sera having IgE for both the two KF LTPs was used as probe. As shown in Figure 12, panel B, full or almost full IgE inhibition results were obtained with the whole KF extracts and the seed ones, whereas the pulp gave either no inhibition (green KF pulp on nAct c 10, Figure 12, panel B) or inhibition values ranging between 18% and 28% (all other pulp/allergen combinations in Figure 12, panel B). These slightly positive results were replicated and could suggest the presence of minimal amount of LTP in the KF pulp not detected by the biochemical methods. The very low inhibition values were anyhow not considered conclusive, and needing a third discriminating proof. We thus performed *in vivo* skin testing with green KF seed and pulp preparations in 21 selected subjects. As reported in Figure 12, panel C, the comparative analysis showed statistically significant different results between green KF seed and pulp preparations, nevertheless some of the patients did react to the pulp as better shown for individual patients in Table 3. As the key values were those obtained on patients having severe clinical reactions on KF ingestion, and high or very high ST and IgE scores when tested with nAct c 10 and nAct d 10 (Table 3, lines 15–21), we concluded that tiny amount of LTP are present in the KF pulp. We thus abandoned the idea of challenging KF clinically allergic patients with the separated KF pulp.

**Table 3:** Diagnostic and clinical profiles of patients enrolled because of clinical allergy to peach and positive or negative kiwifruit LTP test results.

	InterAll APC Code	Age	Gender	ISAC IgE°			Skin test*					DBPCFC°°
				nAct c 10	nAct d 10	nPru p 3	nAct c 10	nAct d 10	Seed**	Pulp**	nPru p 3	Green Kiwi
1	ITROMIDI6320	37	M	Neg	Neg	9.92	Neg	Neg	41.00	74.80	315.30	NR
2	ITROMIDI48581	16	F	Neg	Neg	4.09	Neg	Neg	Neg	Neg	98.34	NR
3	ITROMIDI22317	38	F	Neg	Neg	30.54	Neg	Neg	Neg	Neg	7.40	NR
4	ITLATAM10845	20	F	Neg	Neg	1.10	Neg	Neg	24.00	Neg	125.00	NR
5	ITROMIDI46312	18	F	Neg	Neg	4.42	Neg	Neg	9.20	6.60	24.50	NR
6	ITROMIDI46322	30	F	Neg	Neg	8.13	Neg	Neg	Neg	Neg	142.00	NR
7	ITROMIDI3010	33	F	Neg	Neg	0.75	5.70	Neg	3.20	4.40	97.00	NR
8	ITROMIDI960	48	F	Neg	Neg	1.70	6.50	27.40	5.60	Neg	61.83	NR
9	ITROMIDI2444	45	F	Neg	Neg	4.91	11.50	10.00	ND	ND	139.00	NR
10	ITROMIDI53147	35	F	1.01	0.11	1.50	Neg	Neg	12.80	17.20	54.78	NR
11	ITROMIDI1899	38	M	0.16	0.53	0.22	Neg	Neg	14.00	10.40	49.20	NR
12	ITROMIDI18019	39	F	1.94	1.27	8.91	Neg	Neg	21.90	Neg	71.60	NR
13	ITROMIDI16304	20	F	5.4	7.16	29.08	Neg	Neg	Neg	Neg	53.00	NR
14	ITROMIDI41039	20	F	6.07	5.80	3.38	166.40	75.50	26.00	19.00	134.30	NR
15	ITROMIDI53368	38	F	0.95	0.91	1.19	213.80	148.40	184.80	20.20	30.40	NR
16	ITROMIDI2126	25	F	0.14	0.10	4.67	Neg	Neg	53.70	28.80	83.50	OAS
17	ITROMIDI10971	17	M	21.9	21.40	55.6	309.00	267.80	176.70	36.60	100.40	URT - ANG
18	ITLATAM7614	15	M	0.26	0.46	4.06	76.30	65.00	80.80	52.30	52.60	OAS - ANG
19	ITROMIDI2889	27	F	0.33	0.47	8.79	194.60	256.00	88.00	25.00	215.90	OAS - ANG
20	ITROMIDI1911	32	M	37.12	34.57	12.31	102.20	146.50	156.20	41.00	73.80	GI - URT
21	ITROMIDI1181	22	F	0.13	0.75	4.94	29.50	36.50	25.70	24.00	67.70	GI - URT

APC: Allergome Personal Code used in the InterAll e-record.

° ISAC IgE results are expressed as kU/l.

*Results of skin testing are expressed in mm^2^.

**Seed and Pulp extracts were from green kiwifruit. Subjects 1, 4, 5, having positive ST to seed or pulp preparations were found positive to other kiwifruit LTP allergens, Act d 11, profilins, and Act d 2, respectively.

°° ANG: Angioedema; GI: gastro-intestinal tract symptoms including vomiting and abdominal pain; NR: No reactions; OAS: oral allergy syndrome; URT: Urticaria.

### Defining the tolerability of the green kiwifruit by nPru p 3 positive, nAct c 10/nAct d 10 negative subjects

**Figure 13:**
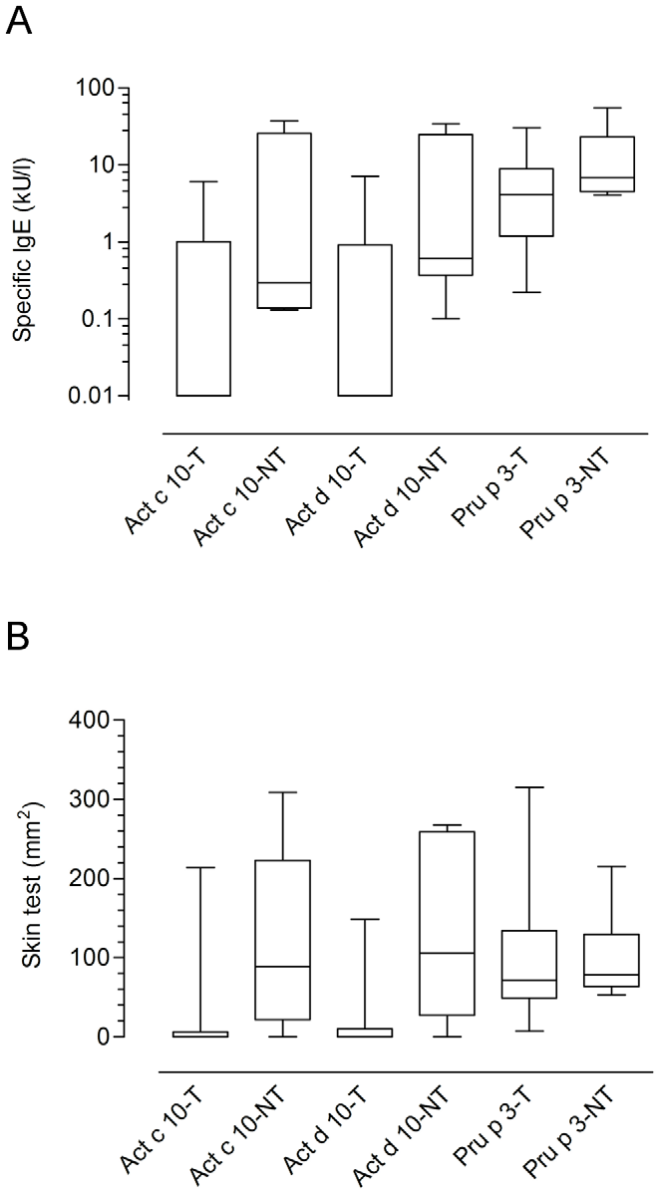
IgE and Skin test result evaluation comparing patients being tolerant or not to green kiwifruit ingestion. NT = Green kiwifruit Non-Tolerant; T = Green kiwifruit Tolerant. Panel A: IgE values obtained testing serum samples for nAct c 10, nAct d 10, and nPru p 3 on ISAC Exp96. The Mann-Whitney test applied to tolerant *versus* non-tolerant patients for each test gave p values as follows: Act c 10-T *vs* Act c 10-NT p = n.n.s.; Act d 10-T *vs* Act d 10-NT p<0.05; Pru p 3-T *vs* Pru p 3-NT p = n.n.s. Panel B: Skin test wheal area values obtained for nAct c 10, nAct d 10, and nPru p 3. Skin test wheal areas have been recorded and expressed in mm^2^. The Mann-Whitney test applied to tolerant *versus* non-tolerant patients for each test gave p values as follows: Act c 10-T *vs* Act c 10-NT p<0.02; Act d 10-T *vs* Act d 10-NT p<0.01; Pru p 3-T *vs* Pru p 3-NT p = n.n.s.

Opposing to the study starting point, where patients used to identify the new KF LTPs were isolated because of their combined clinical and IgE reactivity to KF and peach, and coherently following all the new findings reported above, showing heterogeneous behavior of single subjects toward one or the other LTP, we sought to define the *in vivo* clinical reactivity to green KF in well characterized peach allergic patients. We thus recruited patients having different diagnostic profiles. All enrolled ones had to be nPru p 3 positive with either a positive DBPCFC to peach or a recent severe generalized reaction on peach ingestion. All underwent the tests as reported in Table 3. Among the enrolled ones we had 15 who passed the DBPCFC eating a full KF at the end, and six who clearly showed symptoms after the challenge. As shown in Table 3, the latter were all tested positive for almost all preparations either by *in vivo* or in *in vitro* tests, whereas among the KF tolerant in the majority of the cases the KFs LTP were tested with negative results, but with exceptions, unless positive values were in the lowest range for both ISAC and ST (Table 3). We thus performed a statistical evaluation of test value distributions between the tolerant and the non-tolerant subsets as reported in Figure 13. Unless the number of enrolled subjects was not high, we recorded statistically significant differences when nAct d 10 ISAC IgE values and both KF LTPs ST values were compared, finally showing the valuable diagnostic help of adding KF LTP to the testing to identify patients having a inhomogeneous behavior toward LTPs.

To note that the testing had a positive predictive value for those whose KF avoidance remains mandatory. In addition to those reported in Table 3, seven subjects, who were invited to participate to the study, underwent tests foreseen in the first part, where their diagnostic profile was recorded very similar to that reported for patients 15–21 in Table 3 (data not shown). They declined to undergo the DBPCFC. Six of 7 reported a generalized reaction on KF ingestion sometime in the past, thus they found our nAct c 10 and nAct d 10 positive tests “sufficient to prove their clinically relevant allergy to KF”.

In the attempt to explain why the nAct c 10 or nAct d 10 positive results were not accompanied by a clinical reactivity, we simulated physiological digestion of the two KF LTPs as reported above, and, using the SPHIAa, evaluated the retained capability to induce IgE inhibition. nPru p 3 was comparatively evaluated, as it is known to be resistant to digestion. The results showed that also digestion resistant nAct c 10 and nAct d 10 are still active in the IgE inhibition assay (data not shown).

## Discussion

We herein report for the first time the identification of two new LTPs from the gold and the green KFs and their characterization as clinically relevant allergens. The primary structure of nAct d 10, the one from green KF, has been fully elucidated, being the one of nAct c 10 from gold KF very similar, mainly on the basis of the almost overlapping findings described throughout the present study. The identification process was straight forward as, using a panel of other KF allergens we selected patients being IgE negative for them and having just a positive IgE result for a potentially homologous molecule, namely nPru p 3. This process is highly suggested as leads to a secured evaluation of the newly identified structure, without overlapping positivity as sometimes reported in literature [23]. As the presence of still unknown KF allergens cannot be excluded, patients from the present study should be re-evaluated on the basis of a more comprehensive allergen panel.

Unless we started the identification using subjects having the double clinical reactivity to peach and KF, the first evidence that we were dealing with new allergens not being part of a tight homogeneous group of molecules came by the comparative evaluation of primary structures and the preliminary extended parallel comparative testing with the peach LTP on the 44 peach allergic subjects. The former is the biochemical evidence that Act d 10 is not closely related to anyone of the already known allergenic LTPs. In fact, the observed sequence identities are never higher than 55% and generally fall in the narrow range of 40–55%, whereas more closely related LTPs display higher values. For instance, identities of 97, 91, 88, 87 and 79% are obtained when Pru p 3 sequence is compared with Pru du 3, Pru ar 3, Pru d 3, Pru av 3, Mal d 3 ones, respectively [19]. The alignment of Act d 10 sequence with the five LTPs included in this study (Ara h 9, Art v 3, Cor a 8, Mor n 3, Pru p 3) and the additional fifteen allergenic LTPs listed above shows that seven residues, G31, D45, R46, L53, K54, A67, S83 (Act d 10 numbering) and the 8 cysteine residues, playing a key role in the stabilization of the tertiary structure of the LTP1 proteins, are conserved in all these molecules (data not shown). The analysis of the alignment also shows that, depending on the compared sequences pair, unique combinations of conserved residues occurr. The absence of sequence regions conserved in all the LTP1 suggests that the existence of even one identical epitope, shared by all the allergenic LTP1, is unlikely. It is coincevable that a complete or partial epitope sharing can be found only when closely related LTPs are compared. The observed sequence micro-heterogeneity, mainly found in distantly related LTPs, could be thus the main cause of inhomogeneous epitope patterns producing inhomogeneous LTP IgE recognition as shown at least on the LTP1 included in the present study.

About comparative testing, our data on a huge population where just one LTP, nPru p 3, was tested did not arise such suspicion [4]. Reports from literature leading to characterization of new LTPs are much focusing the description of the new allergen behavior comparing it with Pru p 3, considered the prototype of LTP allergy. We could conclude the same if we stopped our allergen characterization looking at few, highly affected patients as many other reports do on allergens belonging to the same LTP family [19], [24]–[33]. Anyhow, during the time several Authors have observed a certain inhomogeneous behavior of LTPs mainly using IgE inhibition assays or parallel testing with LTPs [19], [24], [29], [33]–[39]. Cited studies, always performed on a limited number of subjects, did not achieve the evidence, we are now reporting, of the highly heterogeneous behavior of LTPs, leading in our opinion to the need of a thorough testing of every single patient with the most comprehensive panel of available LTPs. More emphasis on the topic has been recently brought by Tordesillas et al. [39]. In their study, carried out on a limited number of subjects as well, they included LTPs biochemically-wise quite distant from each other in term of sequences, testing Pru p 3, a very close one, Mal d 3, some as distant as those used in our study (Art v 3, Sin a 3, Tri a 14) and two very remote ones (Par j 1, Ole e 7). Authors finally raised the doubt that the biochemical grouping of allergens can be misleading in the allergy diagnosis. Although Par j 1 and Par j 2 [19] seem definitely placed outside the LTP IgE co-recognized molecules, additional data are required before such conclusion can be drawn at least for Ole e 7, Tri a 14, and Sin a 3.

Regarding molecule to molecule immunological relationships, some studies reported on the need of studying conformational epitopes shared or not by different LTPs [40]–[42]. Using the peptide display library authors searched for identification of peptides working as mimotopes of surface IgE binding regions, giving evidence of the heterogeneity of the molecule surface in LTPs. We tried to give a contribution to the molecule to molecule relationship understanding by extensively studying IgE correlations between paired molecules. IgE correlations have been already reported in few studies in a limited number of subjects, sometimes showing similar results with ours, sometime different [28], [32], [38]. In our opinion this much depend on recruitment selection criteria used to enroll patients in studies. Gadermaier et al. [43] performed IgE correlation experiments on Api g 2, Art v 3, Pru p 3 on a quite large population reporting some findings shown in the present manuscript in a more comprehensive way. In the two studies the sampling bias has been avoided by enrolling patient consecutively without preliminary selection criteria and in a high number. This strategy seems to hold true also for comparing quality of different preparations of the same allergen, as in our case for nPru p 3 from two different providers. Incidentally we have been able to compare them in this high throughput study. Although the large majority of samples were double positive for the two preparations immobilized on the two microarrays, the discrepant results, mainly when high IgE levels are recorded and replicated, require further investigation focusing on both the spotting technology but mainly on the allergen preparation quality.

In the need of profiling patients reporting KF allergy, we used in collaboration with other research groups, Pru p 3 as representative LTP also for KF LTP [44]. After the findings reported in our study we presume that in that study there has been an overestimation of the KF LTP involvement in the patient profiling. Such substitutions should be discouraged as they can lead to over or underestimation of the phenomenon as might be argued for other studies [45]. Nevertheless, unless a high number of LTPs is now available for testing, there are studies claiming the possibility of performing diagnosis and epidemiology of LTP or other allergen sensitizations by using just one representative molecule for each group [46]–[50]. Evidence of a non-homogeneous behavior of profilins as allergens has already been reported by Radauer et al. [51]. Recently the heterogeneous behavior of patients toward Bet v 1 homologous molecules when describing a new Bet v 1-like molecule from green KF, Act d 11 [14], and within the established hevein-like molecule group [52] have been reported. Both studies suggest how the combination of a microarray testing system and panel of molecules might be useful for describing single patient profiles and the overall molecule group picture at the same time. In a very recent paper from Gadermaier et al. [43] this new approach is given for LTPs starting from Api g 2, the celery stalk LTP1. It is evident from this report that a highly selected cohort could drive to wrong conclusions, whereas exploring the behavior in a larger cohort and combining other experimental approaches as in the present study highlights the inhomogeneity of LTP as allergens. Other collaborative studies have been recently accepted for publication showing or reinforcing the demonstration of the valuable use of the multiple homologous molecule approach for Bet v 1-like, tropomyosins, profilins as pan-allergens [53], [54].

Overall we can describe the clinical relevance of our findings as follows: we found non-symptomatic subjects as in previous studies [28], [38], [55], or different clinical pictures to the leading Pru p 3 sensitization as described so far [36], [37]. As shown in several experiments reported in the present study, each LTP shows a different recognition pattern and we could not come to our conclusions without considering such heterogeneity. Thus, level of Pru p 3 IgE cannot account of sensitization toward other LTPs, as recently proposed by Asero et al. [49], whereas the finding of several subjects having isolated IgE positivity to single LTP should be further explored with a broader allergen panel in order to identify whether sources other than peach can account of an IgE sensitization leading to a different LTP subset. At this regard, previously reported data on Art v 3, the mugwort pollen LTP, seem to support this idea [33], [43]. As levels of IgE in case of isolated positivity were never high, we can speculate about single epitope IgE recognition creating the condition for a positive in vitro test but no clinical reactions. Although requiring further studies on a higher number of subjects, our approach leads to allergy test driven decision on what to exclude but, most importantly, on what to leave in patient's diet, we do consider this approach of great clinical relevance as it has never been proposed before. Having robust data coming from a broad positive and negative IgE testing will help the clinical allergist in her/his therapeutic decisions, and hopefully reduce the fear of eating any plant-derived food, typical behavior of LTP allergic patients. The re-introduction of KF under supervision in a clinical setting ready for emergencies is the suggested strategy, mostly in those patients showing discordant *in vivo/in vitro* results as herein reported.

After all, it remains that LTP represent a cross-sectional group of allergens having a patient's related IgE co-recognition. What should be avoided is the interpretation of LTP as part of a given allergenic source in the so called component-resolved diagnosis [26], [29], [44], [45], [56]–[59]. This concept leads back to the allergenic extract-based diagnosis, whereas the allergist needs to be informed on the extension of the IgE recognized homologous molecules and translate that for the patients.

Last important finding of our study is the original distribution of LTP within the KF tissues. LTP is generally reported to be found on the surface of fruits, as in the case of peach [60]. We found a great gap in the presence of the molecule comparing the seeds and the pulp, unless a tiny amount might be found in the latter that we considered enough to exclude the use of KF pulp separated from seed in KF allergic patients. Furthermore, this can justify why in a recent study from us where green KF were evaluated at different ripening stage we did not describe the LTP band [61]. Moreover, the absence of good quantity of the LTP in the pulp raises doubts about the usefulness of the prick-prick test technique as reported negative in some subjects in the present study.

In conclusion, we suggest the use of the microarray testing system bearing panels of homologous molecules as it adds much to our comparative knowledge on biochemical, immunochemical and clinical differences between closely related structures. Clinical allergists might take advantage of missing IgE recognition of one or the other LTP for keeping the specific food in patient's diet, avoiding useless exclusion, generally leading to deterioration of patient's quality of life.

Furthermore, we herein document the need of having the microarray-based testing available for routine allergy diagnosis workup, as it creates the conditions for generating a wealth of data without selection biases and on cohorts otherwise not approachable, as we cannot exclude a different behavior of patients living in different geographical areas. As preliminary depicted in a review by us [3] and more recently discussed on defining the best diagnostic approach to atopic dermatitis patients [62], the most extended and comprehensive panel of molecules will lead us to increase our knowledge on reciprocal IgE recognition of allergenic molecule and of patient's immune profiling, leading to a great improvement of disease management.
